# 

**Published:** 1848-01-01

**Authors:** 


### THE JOURNAL

OF

### PSYCHOLOGICAL MEDICINE

AND

### MENTAL PATHOLOGY.

EDITED BY

FORBES WINSLOW, M.D.

VOL. I.

Surgeon Genl's Office LIBRARY. 22045 Washington, D.C.

LONDON:
JOHN CHURCHILL, PRINCES STREET, SOHO.
MDCCCXLVIII.

We have to apologize to many of our kind friends for the non-appearance of their communications in our present number. Many of the articles—some of great value and deep interest—came after the greater portion of the sheets were in the press. It would be invidious to mention the names of the distinguished members of the profession who have promised to afford the Journal their patronage and support. We have been favoured with letters from nearly all the physicians and surgeons associated with the public and private asylums in the country, and they have, *una voce*, hailed with satisfaction the announcement of the publication of a journal devoted to the investigation of the pathology of the human mind. All have promised us their kind co-operation. We had prepared an elaborate paper, based on the reports of British asylums, for publication, but we were, for want of room, reluctantly compelled to postpone its appearance until the next number of the journal. The following reports have been received:—The Crichton Royal Institution for Lunatics, Dumfries, (for several years;) Annual Report of the Royal Edinburgh Asylum; the Dundee Royal Asylum, (for several years;) Cumberland Lunatic Asylum; Morningside Asylum; Norfolk Asylum; and other works, pamphlets, and communications, which will be noticed in due time. We also have to acknowledge the receipt of several works from Germany and France, on psychological subjects, analyses of which will appear in our forthcoming numbers. Among other works forwarded, we may mention the admirable treatise of Mr. Taylor, "*On Poisons*."

Communications, Books for Review, &c., may be forwarded direct to the Editor, Hammersmith, or to the care of the Publisher.

